# Prognostic significance of ST3GAL-1 expression in patients with clear cell renal cell carcinoma

**DOI:** 10.1186/s12885-015-1906-5

**Published:** 2015-11-09

**Authors:** Qi Bai, Li Liu, Yu Xia, Qilai Long, Jiajun Wang, Jiejie Xu, Jianming Guo

**Affiliations:** Department of Urology, Zhongshan Hospital, Fudan University, Shanghai, 200032 China; Department of Biochemistry and Molecular Biology, School of Basic Medical Sciences, Fudan University, Shanghai, 200032 China

**Keywords:** Clear cell renal cell carcinoma, β-Galactoside α-2,3-Sialyltransferase 1, Prognosis, Nomogram, Overall survival, Disease free survival

## Abstract

**Background:**

Aberrant sialylated carbohydrate synthesis is frequently noted in various cancers. Sialyltransferase ST3GAL-1, which adds a sialic acid in an α-2,3 linkage to Gal β1,3 GalNAc, preforms an important role in modulating cellular behaviors. However, little is known about prognostic significance of ST3GAL-1 in clear cell renal cell carcinoma (ccRCC). In this study, we aimed to investigate the prognostic significance of sialyltransferase ST3GAL-1 and its correlation with clinical outcomes in patients with ccRCC.

**Methods:**

A total of 286 patients who underwent nephrectomy between 2005 and 2007 in a single academic center were recruited. Immunohistochemical staining was performed on tissue microarrays to assess the expression level. Kaplan-Meier method and Cox proportional hazard model were applied to assess the prognostic value of ST3GAL-1. Nomograms were generated as prediction model for overall survival and disease free survival at 5 and 8 years after nephrectomy.

**Results:**

The present results show high expression of ST3GAL-1 is associated with reduced overall survival (*p* = 0.013) and disease free survival (*p* = 0.004). In multivariate cox analyses, ST3GAL-1 was defined as an independent prognostic factor for overall survival (*p* = 0.006) and disease free survival (*p* = 0.001). After incorporation into the University of California Integrated Staging System (UISS) intermediate/high risk group for non-metastatic ccRCC, ST3GAL-1 could further distinguish patient with dismal prognosis (*p* = 0.015 and 0.002 for OS and DFS respectively). The nomograms revealed better predictive accuracy in predicting 5- and 8- year overall survival and disease free survival than the TNM stage alone.

**Conclusions:**

ST3GAL-1 is an independent adverse prognostic factor for recurrence and survival of patients with ccRCC.

## Background

Renal cell carcinoma (RCC) is the most common solid lesion within kidney and accounts for 2–3 % of all cancers in adults [1]. The predominant histologic subtype is clear cell renal cell carcinoma (ccRCC) (70–85 %) [2]. Although most patients with localized tumors can be cured with surgical therapies, 20–30 % of the patients without any evidence of metastasis will develop relapse and/or metastatic RCC in the future [3]. Currently, TNM stage, Fuhrman grade, and Eastern Cooperative Oncology Group performance status (ECOG PS) have been widely used as predictors of clinical outcomes for patients with RCC. Several outcomes prognostic prediction algorithms like University of California Integrated Staging System (UISS), which incorporates TNM stage, Fuhrman grade, and ECOG PS, have been proposed previously to stratify the clinical outcomes after nephrectomy [4, 5]. But the biological nature of ccRCC is kind of complex with an unpredictable course; even tumors with the comparable stage or the same pathological type could show a wide variation in biological behavior and clinical outcomes. The improvement of the predictors of clinical outcomes for RCC is needed [6, 7].

Change in the structure of glycan chains is one of the most shared features of the malignant transformation [8]. The oligosaccharide chains of glycoprotein and glycolipid are often terminated by sialic acids, which play intensely important role in regulating physiologically and pathologically interactions with ligands [9]. In types of cancers, abnormal high level of sialylated tumor associated carbohydrate antigens are frequently noted [10, 11]. The up-regulated sialylation may mediate key pathophysiological events during various steps of tumor progression [12–14]. β-galactoside α-2,3-sialyltransferase 1 (ST3GAL-1), which catalyzes the addition of sialic acid in an α-2,3 linkage to Gal β1,3 GalNAc, preforms important role in modulating cellular behavior. Increasing studies have revealed that the abnormal activity of ST3GAL-1 contributes to the tumor progression [10, 15–17]. However, few studies dedicated to evaluate the expression of ST3GAL-1 and its correlation with ccRCC pathological characters, making the issue elusive.

In this study, we aimed to investigate the prognostic significance of sialyltransferase ST3GAL-1 expression and its correlation with clinical outcomes in patients with ccRCC. ST3GAL-1 expression was assessed by immunohistochemistry, and nomograms integrating ST3GAL-1 with other prognostic parameters were generated to refine individual risk stratification in ccRCC patients.

## Methods

### Patients

We retrospectively collected 286 patients who underwent nephrectomy between 2005 and 2007 at Zhongshan Hospital Fudan University (Shanghai, China). Patient records include TNM stage, Fuhrman grades, histology type, ECOG PS were extracted from the database of the institution, and all the data above were accessible. Other clinicopathological and baseline demographic characters include age, sex, tumor size, and necrosis were also collected retrospectively. Patients with localized RCC (*n* = 271) were treated with either nephron spare surgery or radical nephrectomy. Patients with metastatic RCC (*n* = 15) at diagnosis received cytoreductive nephrectomy followed by interferon-α based immunotherapy. Tumor stage and postoperative histopathological type was determined according to the 2010 AJCC TNM classification [18]. The inclusion criteria were as follows: (1) the histopathological type proved to be ccRCC, (2) all the individuals had no history of anticancer therapy before the nephrectomy, and (3) had no history of other malignant before. If the histopathology was mostly necrosis (>80%) or the morphologic features represent a mixture type of clear cell RCC and other RCC were excluded from the present study. The UISS predictive model was applied to all cases to stratify the risk groups. Most patients underwent regular follow-up every 6 months or earlier for the first 2 years right after the nephrectomy and every 12 months thereafter. Clinical Research Ethics Committee of Zhongshan Hospital, Fudan University had approved the study and granted permissions to access the patient records. Written, informed consent was obtained from each individual enrolled in the study.

### Immunohistochemical staining and evaluation

Hematoxylin and eosin-stained slides had been screened for optimal tumor content before we constructed tissue microarray (TMA) slides. Two cores were taken from the formalin-fixed, paraffin embedded surgical specimens with 2.0 mm diameter punch to make sure each array represented the dominant focus of ccRCC. Immunohistochemical staining was performed on TMA, and the primary antibody was rabbit polyclonal anti-SIAT4A antibody (ab96129, Abcam, Cambridge, MA, USA). The images of stained tissue were captured by the computerized image system composed of an Olympus CCD camera connected to a Nikon eclipse Ti-s microscope. The immunohistochemistry samples were scanned at high-power magnification (×200) and photographed by NIS-Elements F3.2 software. The staining intensity and extent were scored by two independent pathologists who were blind to the clinical outcomes using the integrated optical density (IOD) calculated by Image‐Pro Plus version 6.0 software (Media Cybernetics Inc., Bethesda, MD, USA). The intensity score was graded as 0 (negative), 1 (weak), 2 (moderate), and 3 (strong); the extent score was calculated by the percentage of the positive cells (0–100 %). The staining intensity and extent were then multiplied to generate the expression score ranging from 0 to 300. The score of 210 was selected as the cutoff point of high/low expression by the X-Tile software (Yale University School of Medicine, New Haven, CT, USA).

### Statistical analyses

The correlation between ST3GAL-1 expression and clinicopathological characteristics was analyzed with Fisher’s exact test, χ2 test or t test. Kaplan-Meier method was applied to compare overall survival (OS) and disease free survival (DFS) for groups of low and high expression. Statistical significance was calculated using log-rank test. Multivariate Cox proportional hazard models were used to estimate and test the impact of demographic characteristics, clinical features and ST3GAL-1 expression on overall survival and disease free survival. Hazard ratio (HR) and 95 % confidence interval (CI) were calculated.

The nomograms were generated using R software with “rms” package (R Foundation for Statistical Computing, Vienna, Austria). Selection of variables included in nomograms was based on statistical significance of multivariate analyses, and tumor size was also included in the model as continuous variable. For DFS, we divided T stage variable into T1a and T1b + T2, due to the clinical similarity for metastases after radical nephrectomy [19]. Calibration plots for 5- and 8- year OS and DFS were generated to explore the performance characteristics of the predictive model. The Harrell’s concordance indices (c-indices) were used to measure the prognostic accuracy. All data analyses above were performed using SPSS version 19.0 (SPSS Inc., IL, Chicago, USA) and R software with “rms” package (R Foundation for Statistical Computing, Vienna, Austria). All statistical tests were two sided and considered significant at two-sided *p* <0.05 levels.

## Results

### ST3GAL-1 expression and survival analyses in human ccRCC

**Fig. 1:**
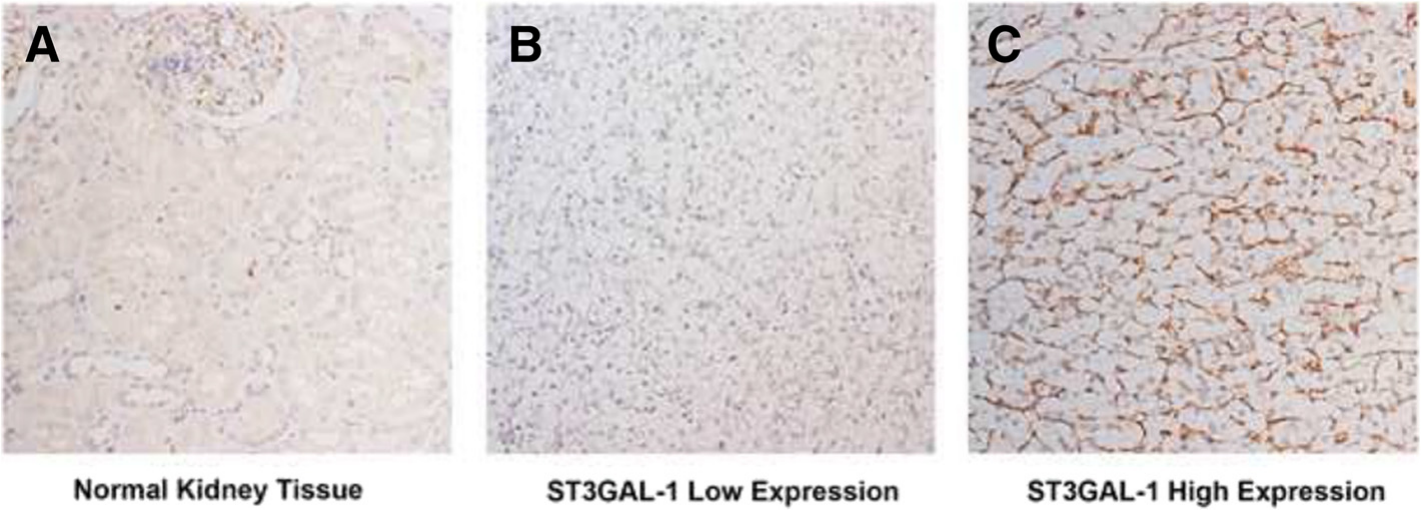
Representative photographs of ST3GAL-1 immunostaining. ST3GAL-1 expression in normal kidney tissue (**a**); Low ST3GAL-1 expression in tumor tissue (**b**); High ST3GAL-1 expression in tumor tissue (**c**). Original magnification: ×200

**Fig. 2:**
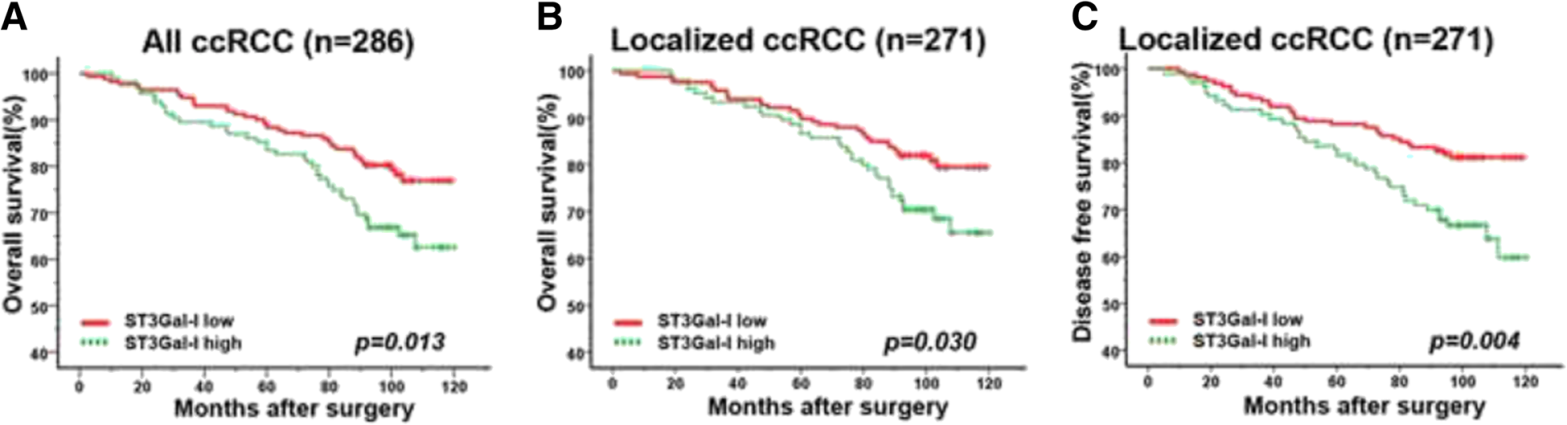
Kaplan-Meier analyses for overall survival and disease free survival of patients with ccRCC according to ST3GAL-1 expression. Overall survival according to ST3GAL-1 expression in all ccRCC patients (**a**); overall survival according to ST3GAL-1 expression in localized ccRCC patients (**b**); disease free survival according to ST3GAL-1 expression in localized ccRCC patients (**c**); p**-**value was calculated by Log rank test, <0.05 was regarded as statistically significant

The representative images of immunostaining were present in Fig. 1. The ST3GAL-1 expression was very low or not detectable in normal kidney tissues (Fig. 1a). The positive staining of ccRCC predominantly appeared in the cell cytoplasm (Fig. 1c). We analyzed a total of 286 patients in the present study. As Table 1 presented, the mean (SD) age was 55.0 (13.0) years old and the median follow up was 99 (range 2.63–120.47) months. Thirteen patients had distant metastasis, and two patients had regional lymph node metastasis at time of surgery. The Kaplan-Meier curves show that patients with high ST3GAL-1 expression level tend to have significantly adverse outcomes for OS (*p* = 0.013, Fig. 2a) than those with low ST3GAL-1 expression patients. For patients with localized ccRCC, the differences were still significant in both OS (*p* = 0.030, Fig. 2b) and DFS (*p* = 0.004, Fig. 2c).

**Table 1:** Associations between ST3GAL-1 expression and clinicopathological characteristics in patients with ccRCC

		ST3gal-1 expression		
Characteristics	All patients	Low	High	p*
Age (years)				0.656
Mean ± SD	55.0 ± 13.0	54.7 ± 13.6	55.4 ± 11.9	
Gender				0.455
Female	90	57	33	
Male	196	115	81	
T stage				0.913
T1	180	106	74	
T2	24	14	10	
T3	79	50	29	
T4	3	2	1	
N stage				1.000
N0	284	171	113	
N1	2	1	1	
M stage				0.102
M0	273	167	106	
M1	13	5	8	
TNM stage				0.334
I	176	105	71	
II	20	12	8	
III	75	49	26	
IV	15	6	9	
Fuhrman nuclear grade grade				0.604
1	31	19	12	
2	216	127	89	
3	37	24	13	
4	2	2	0	
Necrosis				0.652
Absent	249	151	98	
Present	37	21	16	
ECOG PS				0.587
0	198	117	81	
≥1	88	55	33	

Abbreviations: *ECOG PS* Eastern Cooperative Oncology Group performance status, *ccRCC* clear cell renal cell carcinoma

*χ2 test or t test was performed. *p* < 0.05 was regard as statistically significant

### Extension of the UISS (non-metastatic) prognostic model with ST3GAL-1

**Fig. 3:**
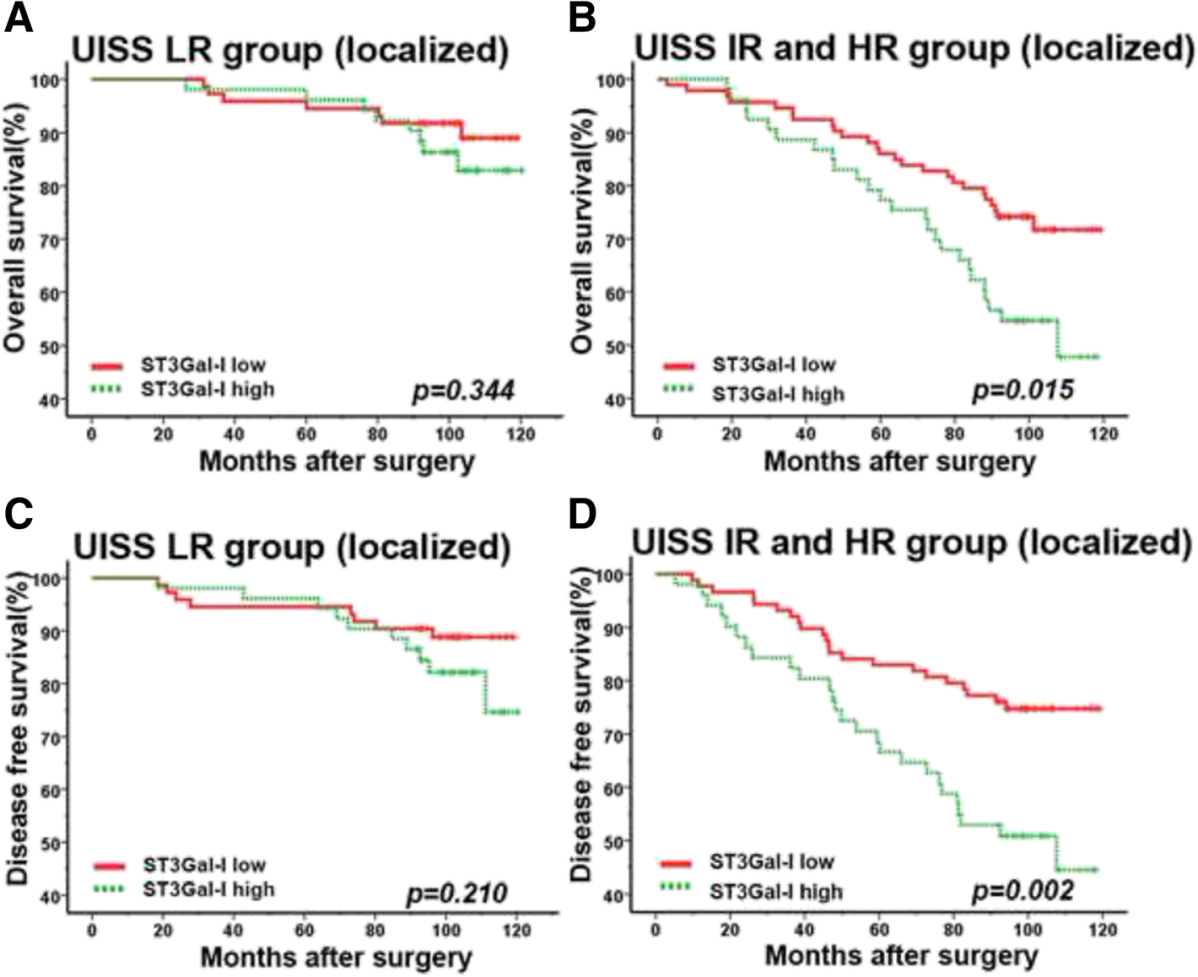
Kaplan-Meier analyses for overall survival and disease free survival of patients in UISS (non-metastatic) subgroups. Overall survival for patients in the UISS (non-metastatic) low risk group (**a**) and intermediate/high risk group (**b**) according to ST3GAL-1 expression; disease free survival for patients in the UISS (non-metastatic) low risk group (**c**) and intermediate/high risk group (**d**) according to ST3GAL-1 expression; UISS: University of California Los Angeles Integrated Staging System; *p* **-**value was calculated by Log rank test, <0.05 was regarded as statistically significant

The UISS (non-metastatic) algorithm performed well in stratifying the risk group for overall survival and disease free survival in our ccRCC cohort (*p* < 0.001). Furthermore, we sought to investigate whether incorporation of high/low ST3GAL-1 expression into UISS outcome algorithm would improve the predictive accuracy by calculating c-index. In the IR/HR group for localized ccRCC, significant difference was found in OS (*p* = 0.015) and DFS (*p* = 0.002) according to the ST3GAL-1 expression (Fig. 3b, d), however ST3GAL-1 failed to further stratify patients with different prognosis in UISS LR patients (Fig. 3a, c). The c-indices of the UISS were 0.65 and 0.63 for OS and DFS respectively, and improved to 0.69 and 0.68 when ST3GAL-1 was added. For patients with metastatic ccRCC, we didn’t use ST3GAL-1 to further refine the UISS (metastatic) algorithm due to the limited individuals.

### Multivariate cox regression analyses

To investigate the prognostic impact of ST3GAL-1 on ccRCC, multivariate Cox regression models were applied (Table 2). In the multivariate cox models, patients with higher ST3GAL-1 expression showed a significantly reduced OS (*p* = 0.006) and DFS (*p* = 0.001) compared with their counterparts. It was also confirmed that TNM stage (*p* < 0.001), Fuhrman grade (*p* = 0.044), Necrosis (*p* = 0.045), and ECOG PS (*p* = 0.047) were independent prognostic factors for OS, and T stage (*p* = 0.003), Fuhrman grade (*p* = 0.020) and necrosis (*p* = 0.015) were independent prognostic factors for DFS in ccRCC.

**Table 2:** Multivariate cox regression analyses for overall survival and disease free survival in ccRCC patients

Variables	Overall survival			Disease free survival		
	HR	95 % CI	p*	HR	95 % CI	p*
TNM stage			<0.001			0.003
III + IV vs I+II	2.76	1.75–4.34		2.13	1.30–3.51	
Fuhrman			0.044			0.020
3 + 4 vs 1+2	1.85	1.02–3.37		2.19	1.13–4.23	
Necrosis			0.045			0.015
Present vs Absent	1.81	1.01–3.25		2.16	1.16–4.00	
ECOG PS			0.047			0.267
≥1 vs 0	1.61	1.01–2.56		1.35	0.80–2.29	
ST3GAL-1			0.006			0.001
High vs Low	1.88	1.20–2.94		2.30	1.40–3.77	

Abbreviations: *ECOG PS* Eastern Cooperative Oncology Group performance status, *CI* confidence interval, *HR* hazard ratio

*Data obtained from the Cox proportional hazards model; *p* <0.05 was regard as statistically significant

### Nomogram for predicting overall survival and disease free survival in ccRCC

**Fig. 4:**
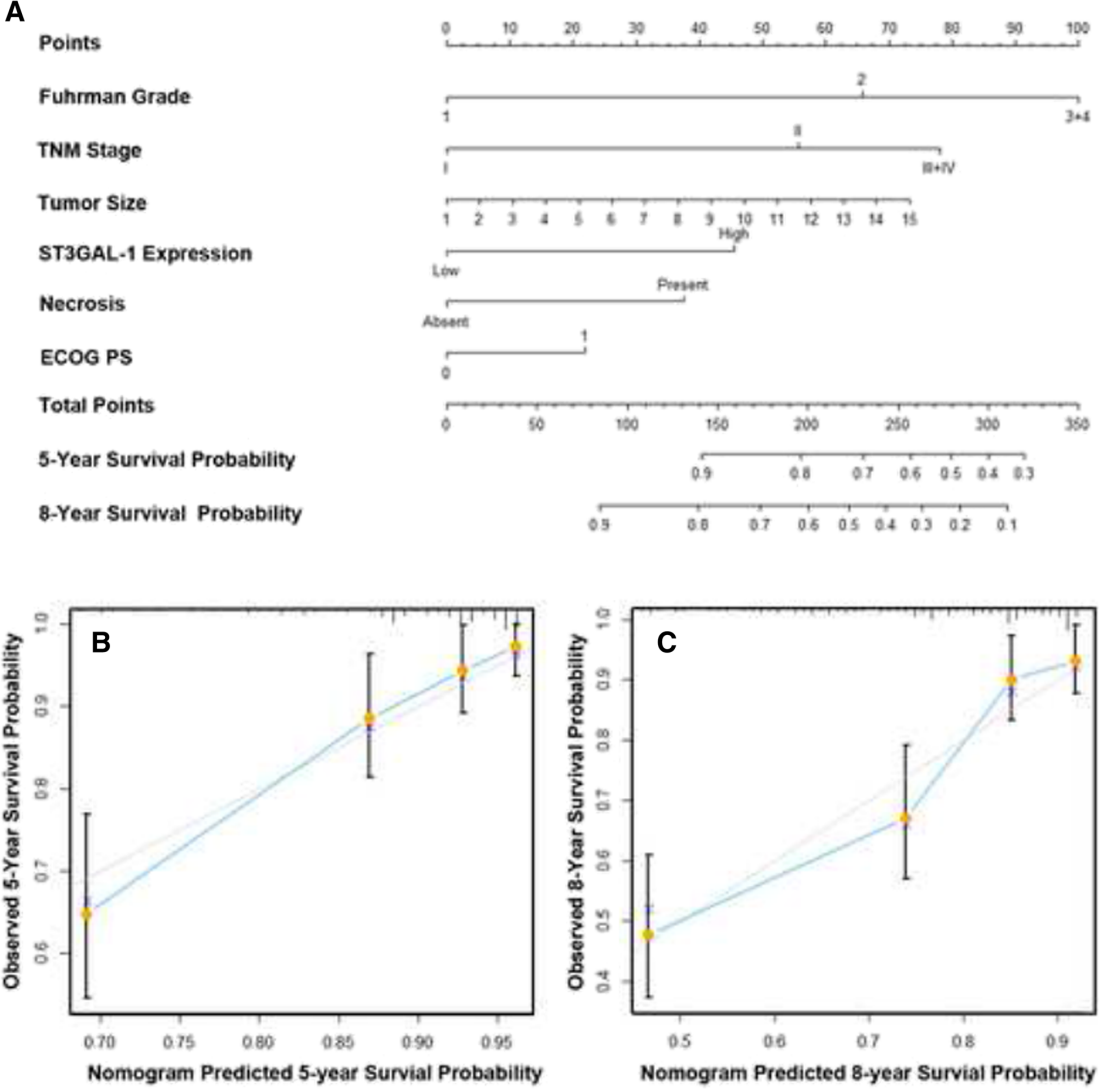
Nomogram for predicting 5- and 8-year overall survival in patients with ccRCC. **a** Nomogram for predicting clinical outcomes integrated with Fuhrman grade (1/2/3 + 4), TNM stage (I + II/III + IV), tumor size (continuous variable), necrosis (absent/present), ECOG PS (0/≥1), and ST3GAL-1 expression (low/high); **b** Calibration plot for nomogram predicted and observed 5-year overall survival rate; **c** Calibration plot for nomogram predicted and observed 8-year overall survival rate. Line of dashes: ideal model, vertical bars: 95 % confident interval. ECOG PS = Eastern Cooperative Oncology Group performance status. Higher total point indicated a more adverse outcomes probability

**Fig. 5:**
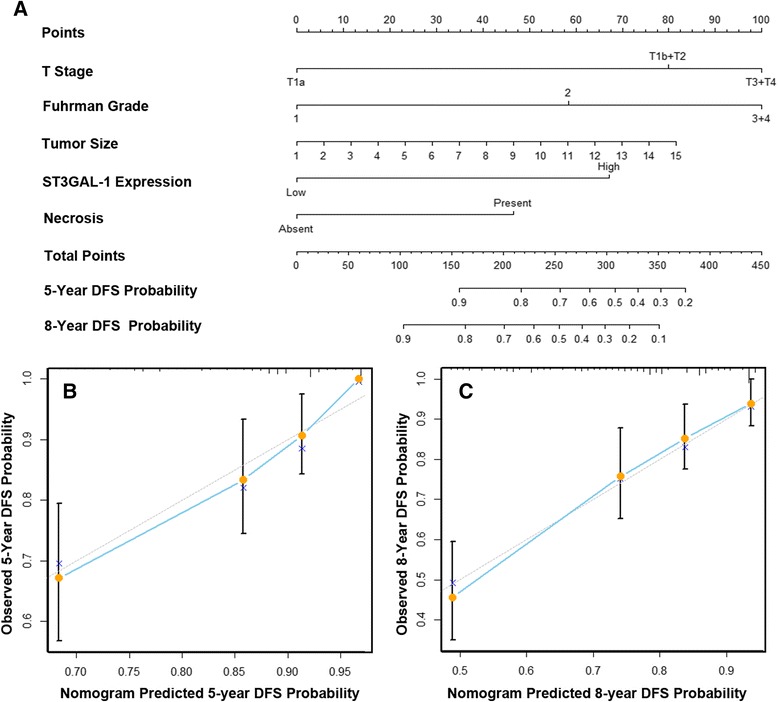
Nomogram for predicting 5- and 8-year disease free survival in patients with ccRCC. **a** Nomogram for predicting disease free survival integrated with T stage (T1a/T1b + T2/T3 + T4), Fuhrman grade (1/2/3 + 4), tumor size (continuous variable), necrosis (absent/present) and ST3GAL-1 expression (low/high); **b** Calibration plot for nomogram predicted and observed 5-year disease free survival rate; **c** Calibration plot for nomogram predicted and observed 8-year disease free survival rate. Line of dashes: ideal model, vertical bars: 95 % confident interval. Higher total point indicated a more adverse outcomes probability

Nomograms were generated to predict OS (Fig. 4a) and DFS (Fig. 5a) at 5 and 8 years after nephrectomy. Total points were calculated to evaluate the clinical outcomes, with higher point indicating more adverse outcome probability. Calibration plots of the nomograms are shown for 5- and 8- year prediction of OS (Fig. 4b, c) and DFS (Fig. 5b,c). The Harrell’s c-indices, which indicated the accuracy of the prognostic model, were 0.753 (95 % CI, 0.701–0.805) and 0.745 (95 % CI 0.690–0.800) for OS and DFS respectively, higher than that of the TMN stage alone (0.703, 95 % CI: 0.647–0.759 and 0.660, 95 % CI: 0.599–0.721).

## Discussion

Glycosylation is quite common in posttranslational modifications. Almost all the cell surface proteins are glycosylated. The aberrant glycosylation, which is aroused by the altered expression and activity of glycosyltransferase, can dramatically affect the glycoprotein and then change the behavior of cell [20–22]. Sialic acids, a family of nine carbon sugars are one of the most important monosaccharide decorating the oligosaccharide chains of several classes of cell surface and secreted glycan molecules. The sialylation of the glycan plays very important role in regulating cellular interaction and signal transduction. The prominent position on cell membrane allows sialoglycan to effectively participate in cell-cell and cell-extracellular matrix interaction, including adhesion, migration, and immune recognition [23]. Hypersialylation of glycan in tumor cells is associated with metastatic behaviors including invasion and enhanced cell survival and correlates with a poor prognosis for patients with cancer [24].

It is now well-established that altered activity of sialyltransferase is one of the key mechanisms to trigger aberrant sialylation of glycans and expression of specific tumor associated carbohydrates in cancer [24]. However, researches focus on the sialylation glycans and sialyltransferase in ccRCC is now devoid. So in this study, we put our focus on the sialyltransferase ST3GAL-1, which adds a sialic acid in an α-2,3 linkage to Gal β1,3 GalNAc. In the study, we have revealed the dismal role of ST3GAL-1 and its impact on patients with ccRCC by survival analyses. The present results showed that ccRCC patients with higher level of ST3GAL-1 expression tend to have unfavorable clinical outcomes than the counterparts. We also demonstrated that incorporating ST3GAL-1 into UISS (non-metastatic) could refine the algorithm prediction. In multivariate cox regression model, it was proved that ST3GAL-1 was an independent prognostic factor for OS and DFS. Unexpectedly, the N stage in multivariate analyses was not considered an independent prognostic factor, which was probably due to the limited number of N1 individuals (*n* = 2).

Several studies have elucidated the association of ST3GAL-1 with tumor progression. Overexpression of ST3GAL-1 has been reported in types of tumors like colon and breast cancers [10, 17]. P. Videira found mRNA level of ST3GAL-1 was significantly higher in malignant bladder tumor [15]. It has also been confirmed that abnormal ST3GAL-1 activities could involve in malignant cell transformation and tumor progression. In breast carcinoma, constitutive ST3GAL-1 expression contributed to an unfavorable prognosis [16]. In colorectal carcinoma, ST3GAL-1 expression was associated with lymph node metastasis [10]. Previous studies have reported that EMT, which is essential for tumor progression, is associated with altered expression of sialoglycans [25]. Up-regulation of sialyltransferase and following expression of sialoglycan is an important step underlying the migratory phenotype during EMT. Moreover, the proto-oncogene, c-myc could up-regulate the ST3GAL-1, while the metastasis suppressor gene nm23-H1 plays an opposite role [25, 26], indicating the protumoral role of ST3Gal-1 in the development and progress of tumors.

Selectins, receptors for sialyl Lewis^x^ (sLe^x^) and sialyl Lewis^a^ (sLe^a^) expressed on endothelial and blood cells play an important role in mediating malignant cells dissemination [12, 27]. Recent reports have been indicative of ST3GAL-1’s responsibility for the high expression of the sialoglycans sLe^x^ and sLe^a^ [25, 28]. Increased sLe^x^ was reported in liver metastasis of colorectal cancer, and was associated with poor patient survival and early recurrence in colorectal carcinoma [27, 29]. The interactions between selectins with sLe^x/a^ result in tumor cell adherence to blood vessels, thus facilitate extravasation into surrounding tissue, and trigger angiogenesis [30]. After tumors invade into vasculature, malignant cells with higher ST3GAL-1 were more likely to aggregate with platelets, and eventually lodge in the small vessels and distant organs [31]. Thus, it is conceivable that elevated sLe^x/a^ antigen synthesis caused by ST3Gal-1 in the tumor favors tumor cells to bind to selectins and then extricate from the primary tumor enter into blood stream and promote metastasis formation. This could give a possible explanation for our finding that patients with higher ST3GAL-1 expression are more likely to suffer recurrence and metastasis in the future, and ST3GAL-1 expression is also an independent risk factor for the disease free survival in ccRCC. Nowadays, increasing researches focusing on the sialylated glycan pathways have show a remarkable effectiveness for tumor therapy in preclinical and clinical studies [32–34], which might be suggestive that targeting sialyltransferase could be a novel approach to deal with RCC some day.

At present, the role of ST3GAL-1 in ccRCC is far from fully elucidation and need further exploration. Limitations of our study are the retrospective design and relatively small study cohort. A multicenter and prospective study is needed to validate the results. Moreover, other sialyltransferase like ST3GAL-3, ST3GAL-4 were not analyzed in the present study and merit further research.

## Conclusion

In conclusion, we have confirmed ST3GAL-1 is correlated with unfavorable outcomes and could be used as an independent prognosticator in patients with ccRCC. We also developed nomograms for OS and DFS, which could give a better prediction for patients with ccRCC after surgery.

## Abbreviations

ST3GAL-1: β-Galactoside α-2,3-Sialyltransferase 1
RCC: Renal cell carcinoma
ccRCC: Clear cell renal cell carcinoma
OS: Overall survival
DFS: Disease free survival
UISS: University of California Integrated Staging System
AJCC: American Joint Committee on Cancer
EMT: Epithelial-to-mesenchymal transition
TMA: Tissue microarray
C-index: Concordance index
CI: Confidence interval
